# Underutilized Mexican Plants: Screening of Antioxidant and Antiproliferative Properties of Mexican Cactus Fruit Juices

**DOI:** 10.3390/plants10020368

**Published:** 2021-02-14

**Authors:** Elda M. Melchor Martínez, Luisaldo Sandate-Flores, José Rodríguez-Rodríguez, Magdalena Rostro-Alanis, Lizeth Parra-Arroyo, Marilena Antunes-Ricardo, Sergio O. Serna-Saldívar, Hafiz M. N. Iqbal, Roberto Parra-Saldívar

**Affiliations:** Tecnologico de Monterrey, School of Engineering and Sciences, Monterrey 64849, Mexico; elda.melchor@tec.mx (E.M.M.M.); a00812589@itesm.mx (L.S.-F.); jrr@tec.mx (J.R.-R.); magda.rostro@tec.mx (M.R.-A.); A00812589@exatec.tec.mx (L.P.-A.); marilena.antunes@tec.mx (M.A.-R.); sserna@tec.mx (S.O.S.-S.)

**Keywords:** Underutilized Mexican plants, Cactus fruits, Antioxidant activities, Antiproliferative properties

## Abstract

Cacti fruits are known to possess antioxidant and antiproliferative activities among other health benefits. The following paper evaluated the antioxidant capacity and bioactivity of five clarified juices from different cacti fruits (*Stenocereus* spp., *Opuntia* spp. and *M. geomettizans*) on four cancer cell lines as well as one normal cell line. Their antioxidant compositions were measured by three different protocols. Their phenolic compositions were quantified through high performance liquid chromatography and the percentages of cell proliferation of fibroblasts as well as breast, prostate, colorectal, and liver cancer cell lines were evaluated though in vitro assays. The results were further processed by principal component analysis. The clarified juice from *M. geomettizans* fruit showed the highest concentration of total phenolic compounds and induced cell death in liver and colorectal cancer cells lines as well as fibroblasts. The clarified juice extracted from yellow *Opuntia ficus-indica* fruit displayed antioxidant activity as well as a selective cytotoxic effect on a liver cancer cell line with no toxic effect on fibroblasts. In conclusion, the work supplies evidence on the antioxidant and antiproliferative activities that cacti juices possess, presenting potential as cancer cell proliferation preventing agents.

## 1. Introduction

According to a recent report by the World Health Organization (WHO), cancer is a collection of diseases that is the second leading cause of mortality worldwide. The condition starts in any organ/tissue of the body and spreads beyond its initial boundaries to invade adjoining tissues until eventually it reaches other organs and tissues. The uncontrolled stage of this collection of illnesses is called metastasis and is a major cause of death. Cancer disease cause of death globally accounted for an estimated 9.6 million deaths in 2018, which could have increased by 50% to 15 million by 2020 [1]. The tissues most commonly affected were lung, breast, colorectal, prostate, skin, and stomach. Liver cancer alone caused 782,000 deaths [1]. In order to decrease the mortality rate, efforts should focus on preventing or treating cancer at its early stages. Mexican plants have been proven to act as natural antioxidant products as well as providing anticancer activities [2]. Cactaceae plants are a group of uncommon species worldwide that grow in arid areas. Mexico is home of 518 species of which 47.7% are endemic [3]. Cacti fruits are highly valued in the region for their chemical composition which grants them their distinctive organoleptic properties such as color and taste [4]. Few studies detail the chemical characterization of cacti fruits, and their bioactive properties. 

**Figure 1:**
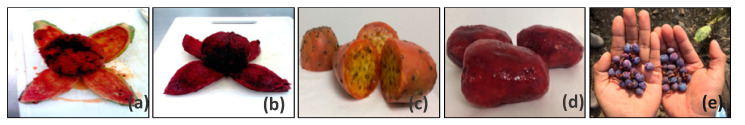
Cactus fruits (**a**) *Stenocereus pruinosus* yellow fruit (SY), (**b**) *Stenocereus pruinosus* red fruit (SR), (**c**) *Opuntia ficus-indica* yellow fruit (OPY), (**d**) *Opuntia ficus-indica* red fruit (OPR), (**e**) *Myrtillocactusgeomettizans* fruit (MG).

Fruits from the *Stenocereus* genus (pitayas) can be harvested semi-annually from April to May and September to October. They are ovoid in shape, pigmented, and possess small thorns [5] (Figure 1). Partial characterizations of pitaya fruits have shown that they contain betalains, flavonoids, and phenolic compounds (caffeic, ferulic, and *p*-coumaric) [3,6]. Their uses have been reported against insect bites, rheumatism, hemorrhages, and gastrointestinal issues [3,7].

Prickly pears are oval shaped fruits commonly referred to as “tunas” (Figure 1) and are extensively distributed throughout Mexico [8]. They have been known to contain flavonoids, phenolic acids (caffeic, coumaric, vanillic, among others), betalains, ascorbic acid, fatty acids, lignans, and sterols [3,6]. Additionally, they possess a variety of health benefits, yet not restricted to a high antioxidant capacity [9], including cytotoxic activity [10,11], and an antiproliferative effect on cancer colon (HT29/Caco-2) and prostate cancer cell lines (PC-3) in vitro [12,13]. 

*Myrtillocactus geomettizan* produces cacti fruits called garambullo, also known as the berry cactus, which ripens from May to July. Garambullo fruits are 1.5 cm in length, globular, and purple (Figure 1) [14]. The berry cactus fruits contain flavonoids, betalains, ascorbic acid, and many phenolic acids (caffeic, gallic, vanillic) [3,6] with associated health properties such as the improvement of renal functions in rats as well as decreased glucose and cholesterol levels in blood [14]. 

There are only a few characterization studies about the Cactaceae family and their fruits. The exploration of nature as a source of novel compounds to treat chronic diseases, such as cancer, is growing. Considering the above properties of underutilized Mexican plants, herein, we report the chemical characterization and compositional analyses of five clarified juices extracted from different cacti fruits (*Stenocereus* sp., *Opuntia* sp. and *M. geomettizans*). The scientific rational behind this study was to test and report the correlation between antioxidant capacity and antiproliferative properties of Mexican cactus fruit juices. Furthermore, we studied the in vitro bioactive potential of in-house extracted juices from different cacti fruits against cancer cell lines and a normal cell line, with the aim of proposing their medicinal valorization.

## 2. Results and Discussion

### 2.1. Total Soluble Solids, Betacyanin, Betaxanthin Content, and Antioxidant Activity

There are few reports quantifying the chemical content of the cactus fruits here mentioned in order to establish a comparison. The clarified juices obtained had low values around 0.2 °Brix, of soluble solid content (Table S1). The juice of Pitaya (*Stenocereus pruinosus*) reported 9.8 °Brix [15], a higher value of the total solids than in SY (0.2 °Brix) and SR (0.2 °Brix). One possible reason for the discrepancy between the results from this paper and those in the literature is a difference in the procedure used to obtain the clarified juices, as showed in the Supplementary material.

The content of betalains is shown in Table 1. The OPR clarified juice had the highest concentration of betacyanins of the clarified juices (403.56 ± 1.41 µg/g of FS-fresh sample). The analysis of the different *Opuntia* fruits showed similar betacyanin compositions to that of *Opuntia robusta*, that goes by the common name of Tapón, reported in the literature (300.5 ± 8.8 µg/g FS) [12]. Regarding the betaxanthin concentrations, the SR clarified juice had the highest value (404.59 ± 2.33 µg/g of FS) followed by the OPR juice (263.24 ± 36.36 µg/g of FS), and the *Opuntia rastrera* juice (86.2 ± 22.3 µg/g FS) [12]. Betalains are water soluble which is why a higher content was observed in the clarified juices than in the pulps. A similar phenomenon was observed in freeze-dried cherry [16]. The clarified juice of *Myrtillocactus geomettizan* had less betacyanins (103.50 ± 0.01 µg/g FS) and betaxanthins (45.76 ± 0.42 µg/g FS), and even betacyanins in the fruit pulp (36.9 ± 3.7 µg/g FS) [17]. This may be due to the stage of maturity and geographical origin of the cactus berries [18]. 

Using the Folin–Ciocalteu method, the MG clarified juice (138.38 ± 0.14 mg GA/ 100 mL of FS) had the highest antioxidant content followed by the OPR clarified juice (111.72 ± 0.36 mg GA/100 mL of FS). A similar behavior to that of the MG clarified juice has been reported in red fruits such as raspberry *Rubusidaeus L*. (148.9 ± 0.45 mg GA/100 mL FS) [19]. The above results could be explained by the resulting effect of a wide range of structures such as monophenols, catechols, pyrogallols, phloroglucinols, resorcinols, p-hydroquinols, naphthols, anthracenes, flavonoid aglycones, glycosides, hydroxycoumarins, aminophenols [20], and vitamins as dehydroascorbic acid [21] that are detected by this technology.

The ABTS method corroborated the antioxidant results from the Folin–Ciocalteu method; the MG clarified juice (3123.77 ± 26.15 µmol TE/100 g FS) had the highest antioxidant content followed by the OPR clarified juice (1097.35 ± 20.20 µmol TE/100 g FS). Red fruits reported higher antioxidant activity through the DPPH procedure; OPR clarified juice being the highest (1115.25 ± 56.46 µmol TE/100 g of F) followed by SR juice (854.601 ± 17.60 µmol TE/100 g of FS). A similar trend was observed in the FRAP results of red fruits. However, the SR clarified juice had the highest antioxidant effect (2744.48 ± 42.16 µmol TE/100 g of FS). Antioxidants assays are classified in electron transfer (ET) methods such as ABTS, FRAP, and DPPH and hydrogen atom transfer (HAT) like methods such as ORAC [22]. The cacti fruits complex matrix contains a variety of chemical compounds that cause their antioxidant properties. In order to obtain an accurate understanding of the antioxidant capacity of cacti fruits and to be able to compare results across different labs with varying conditions, multiple assays with distinct mechanisms must be run. Nevertheless, juices from passion fruit (176.42 ± 23.40 µmol TE/100 mL FS) and lemon juice (56.75 ± 26.63 µmol TE/100 mL FS) [23], demonstrated a lower antioxidant capacity than all clarified juices analyzed here.

Recent studies have found that the flowers also have phenolic compounds, such as *Artocarpus lakoocha Roxb* [24]. For this reason, it will be interesting to study the extract of flowers of *Stenocereus pruinosus*, *Opuntia ficus-indica*, and *Myrtillocactus geomettizans* in future works.

### 2.2. Phenolic Composition Analysis

Due to their availability and cytotoxic activity, MG and OPY were analyzed by HPLC in order to detect the content of *p*-coumaric acid, gallic acid, caffeic acid, and vanillic acid in each clarified juice, using each phenolic acid’s respective standard. Figures S2–S5 displays the HPLC profile of the MG and OPY clarified juices. The results indicated a high content of *p*-coumaric acid (60.60 ± 0.25 mg/L of FS) in MG clarified juice. The OPY clarified juice had a high amount of gallic acid (21.75 ± 0.75 mg/L FS) as well as *p*-coumaric acid (16.85 ± 1.02 mg/L FS) (Table 2). 

The HPLC analysis of the phenolic acids previously mentioned in *Opuntia joconostle* reported significant amounts of protocatechuic and caffeic acid. Vanillic acid was not detected in the whole fruit [25]. Additionally, *p*-coumaric and caffeic acid derivatives were detected in *Opuntia ficus-indica* by chromatography coupled to high resolution time of flight mass spectrometry (UPLC-QTOF-MS) [26], as the present work demonstrated. In the literature, *Myrtillocactus* was reported to have a higher amount of gallic acid than caffeic acid [18], in agreement with the present work.

### 2.3. Cell Viability Assay

**Figure 2:**
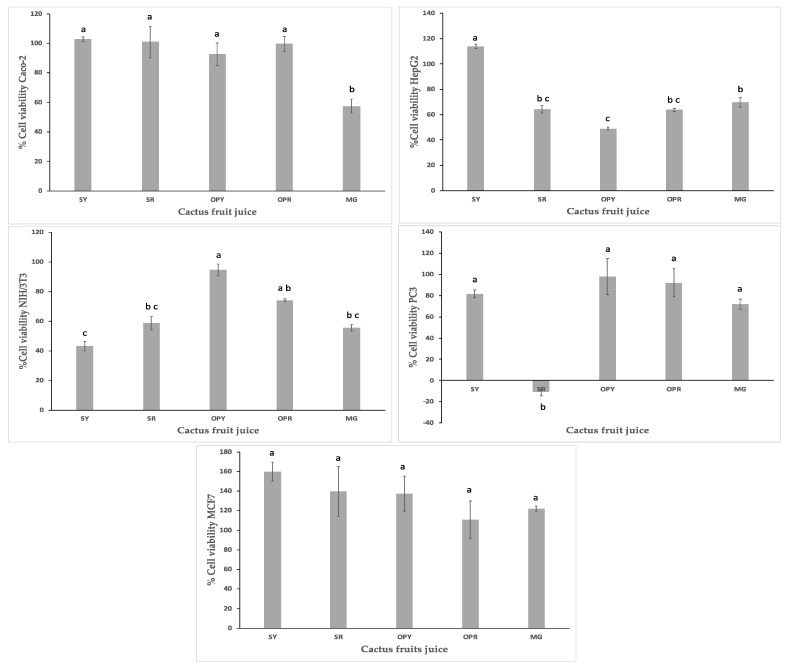
Cell viability of Caco-2, HepG2, NIH/3T3, PC3, and MCF7 cell-lines treated with cactus clarified juices at 2% *v*/*v*. Cell viability was expressed in terms of percentage of living cells relative to the non-treated control. Results were expressed as means of triplicate experiments and error was expressed as Standard error of mean (SEM). Different lowercase letters (a–c) indicate a statistical significance difference (*p* < 0.05). *Stenocereus pruinosus* yellow fruit (SY), *Stenocereus pruinosus* red fruit (SR), *Opuntia ficus-indica* yellow fruit (OPY), *Opuntia ficus-indica* red fruit (OPR), and *Myrtillocactus geomettizans* fruit (MG).

A fast screening of the cytotoxic assay was performed, using a single concentration of the clarified juices of 2% (*v*/*v*) in order to identify the species with the highest cytotoxicity on the cancer cell lines while affecting the normal cell line as minimally as possible. The results showed, HepG2 cells were more sensitive to the clarified juices of OPY, OPR, SR, and MG with cell viability percentages of (49.02 ± 1.32), (63.82 ± 1.08), (64.35.78 ± 2.84), and (69.75 ± 3.70) respectively, compared to the Caco-2 cell line which was only affected by the MG clarified juice with cell viability percentage of (57.50 ± 4.58). All clarified juices except the SR one had a similar effect on the PC3 cell line. The cell viability percentage of the SR clarified juice was not detected in the PC3 cell line, the blank had high values of absorbance, leading to negative cytotoxicity percentages. The antiproliferative effect of the five clarified fruit juices on MCF7 could not be demonstrated as the cell viability percentage was more than 100 percent. The NIH/3T3, normal cell line was used as a control and the SY, MG, and SR clarified juices diminished the cell line proliferation with percentages of (43.15 ± 3.27), (55.68 ± 2.09), and (58.59 ± 4.56) respectively (Figure 2). A future investigation may be performed in order to evaluate the dose and time-dependence of the cactus juices with the potential on cytotoxicity.

In vitro studies of juices extracted from fruits of *Opuntia ficus-indica* showed antiproliferative effects on hepatic cancer cells while no effect on normal fibroblast viability [12], corresponding with the findings of the present work. In order to understand if phenolic acids were responsible for the potential antiproliferative effect of clarified juices, the individual phenolic acids measured by HPLC analysis were calculated in µg/100 µL in the clarified juice at 2% in the cell proliferation assay. This calculation showed that the concentration of the phenolic acids previously mentioned was too low to have significantly contributed to the antiproliferative effect observed (Table S3). Further investigation to detect specific compounds such as quercetin, isorhamnetin, kaempferol, and betalains by HPLC is necessary to prove that the antiproliferative effect above described was due to the phenolic compounds. Evidence of these compounds has been reported in cactus juices. The fruit juices of *Stenocereus pruinosus* and *Stenocereus stellatus* were evaluated by HPLC-DAD-ESI/MS, to quantify the amounts of quercetin 3-*O*- rutinoside, kaempferol hexoside, isorhamnetin hexoside, isorhamnetin 3-*O*- glucoside, nine betacyanins, and two betaxanthins [6,15]. Isorhamnetin, quercetin, conjugated phenolic acids, indicaxanthin and coumarins were observed using UPLC-QTOF-MS in three *Opuntia ficus-indica* juices [26]. Quercetin was found in the cactus berry (*Myrtillocactus geometrizans*) fruit at different maturity stages before and after storage by HPLC-DAD [18]. The antiproliferative activities of flavonoids and betalains have been reported in extracts of *Opuntia robusta* and *Opuntia ficus-indica* fruit juices as they diminished human colorectal cancer cell line HT29 proliferation. The antiproliferative compounds identified were betacyanins, ferulic acid, and isorhamnetin derivatives [13]. In order to evaluate the therapeutic potential of the clarified juices for cancer, the molecular mechanism should be investigated and elucidated on normal and cancer cell lines of the same tissue. Previous studies have demonstrated the effect of plant extracts on proliferation, morphology, and cell death in MCF-7 breast adenocarcinoma and non-carcinogenic MCF-12A cell lines, where MCF-7 cell line was more susceptible to plant extracts exposure [27].

### 2.4. Principal Component Analysis (PCA)

**Figure 3:**
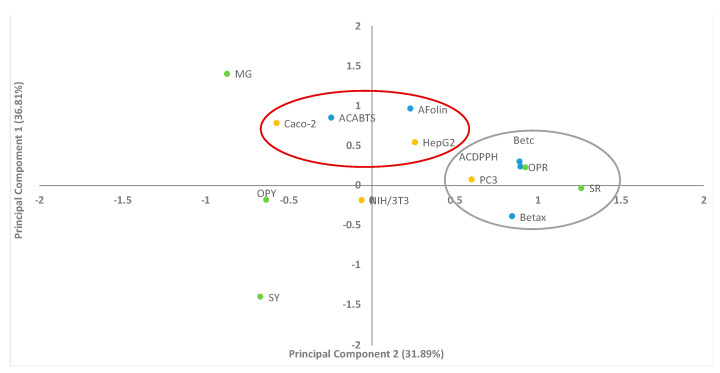
Distribution of five clarified juices along principal components 1 and 2 using nine variables. Betacyanins (Betac), betaxanthins (Betx),antioxidant activity by ABTS method (ACABTS), antioxidant activity by DPPH method (ACDPPH), total phenolic composition (AFolin), fibroblast cell line (NIH/3T3), colon cancer cell line (Caco-2), hepatic cancer cell line (HepG2), prostate cancer cell line (PC3) *Stenocereus pruinosus* yellow fruit clarified juice(SY), *Stenocereus pruinosus* red fruit clarified juice (SR), *Opuntia ficus-indica* yellow fruit clarified juice (OPY), *Opuntia ficus-indica* red fruit (OPR) clarified juice, and *Myrtillocactus geomettizans* fruit (MG) clarified juice. The oranges circles are cancer cell lines, the blue circles are the analytical methods and green circles are the clarified juices.

In this study, PCA was used to correlate nine experimental variables: content of betacyanins and betaxanthins, antioxidant activity by both ABTS and DPPH methods, total phenolic composition by Folin–Ciocalteu assay according to cytotoxic activity of each clarified juice on HepG2, Caco-2, PC3, and NIH/3T3. The MCF7 cell line was not considered in the PCA analysis due to its high cell viability percentage and non-significant difference in all clarified juices. FRAP activity was not included in the PCA plot due to missing experimental results from MG fruit because it was unavailable during the analysis, due to the time at which it was collected. In Figure 3 we identified two components, principal component 1 (cell viability of cancer cell lines HepG2, Caco-2 which have a correlation with total phenolic composition by Folin–Ciocalteu assay and antioxidant activity by ABTS) and principal component 2 (betacyanins and betaxanthins which strongly correlated with antioxidant activity by DPPH and prostate cytotoxic activity on the PC3 cell line with red cactus fruits juices, SR and OPR). The variance of the data for the Principal Component 1 (PC1) was 36.81% and for the Principal Component 2 (PC2) was 31.89% of the variance in the data. The two principal components contributing to 68.7% of the total variance of the results. Based on PCA, the cell viability of the NIH/3T3 cell line was not correlated in either of the two groups. It is a normal cell line that was not observably influenced by the chemical content of the clarified juices in the grouping. Compared to cancer cell lines, PC3 was influenced by the chemical content of betalains and antioxidant compounds measured by DPPH; whilst Caco-2 and HepG2 were influenced by chemical content measured by ABTS and Folin–Ciocalteu methods. OPY, MG, and SY were excluded from principal component 2, due to their lower content of betalains. The PCA analysis evidenced that red cactus fruits showed a higher content of betalains which positively correlated with antioxidant activity by the DPPH method as shown in Table 1. The data is presented by two or three principal components defined as a linear combination and correlation between each other, therefore, it reveals clusters of the observed variables in terms of their similarities and dissimilarities [28]. However, additional investigation is needed in order to demonstrate SR antiproliferative activity on the PC3 cancer cell line. Previous investigations have used PCA to explain attributes of the sample and generate a global analysis of results; for example, to correlate the total phenolic content or flavonoids with antioxidant activity by hydrogen peroxide (H_2_O_2_), hydroxyl (·OH), peroxyl (ROO·) and ABTS radicals from *Opuntia elata* (Arumbeva) fruit extract [29]. Clear correlations were evidenced between total phenolics, fatty acids, phenolic compounds, and antioxidant activity of *Opuntia ficus-barbarica* A. Berger fruit pulp and seed oil harvested at different times [30]. 

## 3. Materials and Methods 

### 3.1. Chemical and Reagents

2,2-Diphenyl-L-picryl-hydrazyl and sodium carbonate were purchased from Sigma Aldrich (Steinheim, Germany). 2,2′-Azino-bis (3-ethylbenzothiazoline-6-sulfonic acid) (ABTS), Folin & Ciocalteu’s Phenol Reagent, potassium persulfate, *p*-coumaric acid (concentration ≥ 98%), caffeic acid (concentration ≥ 98%), vanillic acid (concentration ≥ 97%) were purchased from Sigma-Aldrich (St. Louis, MO). Disodium phosphate and potassium chloride were acquired from Productos Químicos Monterrey S.A. de C.V. (Nuevo Leon, Mexico). was purchased from Productos Quimicos Monterrey S.A. de C.V. (Nuevo Leon, Mexico). Iron (lll) chloride hexahydrate and gallic acid monohydrate were obtained from Sigma Aldrich (Shanghai, China), Glacial acetic acid and methyl alcohol were purchased from Tedia High Purity Solvents (Fairfield, OH), hydrochloric acid was purchased from CTR Scientific (Nuevo Leon, Mexico) and 2,4,6-tris(2-pyridyl)-s-triazine (TPTZ) was from Sigma Aldrich (Buchs, Switzerland). The milli-Q water purification system was used to filter the water that was used to perform the procedures (Q-POD, Darmstadt, Germany). Potassium phosphate monobasic was acquired from Sigma-Aldrich (Tokyo, Japan). Sodium chloride was bought from Desarrollo de Especialidades Químicas, S.A. de C.V. (Nuevo Leon, Mexico). 

### 3.2. Production of Clarified Juice

#### 3.2.1. Preparation of Pitaya, Garambullo, and Tuna Pulps

Around, 3 kg of *Myrtillocactus geometrizan* fruit (MG) and 5 kg of yellow prickly pear *Opuntia ficus-indica* (OPY) from Ahualulco (San Luis Potosí, México); 5 kg of yellow pitaya *Stenocereus pruinosus* fruit (SY) and 5 kg of red pitaya *Stenocereus pruinosus* from Ahuatlán (Puebla, México); and 5 kg of red prickly pear *Opuntia ficus-indica* fruits (OR) from San Nicolás (Nuevo León, México) were refrigerated, while ensuring not handling for longer than 48 hours after being gathered. The fruits were washed with tap water and Extran MA05 (Merck, Item 1400001403, Lot Mx1400005004, Estado de Mexico, Mexico). Afterwards, the spines and peels were detached manually. Ultimately, the pulp, and seeds were separated using a juice extractor (Model TU05, Turmix ML, Estado de México). Seedless pulp was obtained from this procedure and its moisture was measured [31].

#### 3.2.2. Production of Clarified Juice

The following procedure was carried out in the dark. The previously acquired seedless pulp (30g) was centrifuged (4000 g, 4 °C, 10 min, Model SL 40R, Thermo Fisher Scientific, Langenselbold, Germany) in 50 mL polypropylene conical tubes (Corning®, Tewksbury, MA, USA). 30 g of pulp of each fruit (SY, SR, OPY, OPR, and MG) was prepared to obtain clarified juice as shown in Figure S1. The supernatant was strained though 150 mm of Whatman paper grade 4 (item 1009150, GE Healthcare Life Sciences, Little Chalfont, UK), the supernatant of this second filtration was considered the clarified juice. Water was not added to the clarified juices.

### 3.3. Total Soluble Solids

The total soluble solids (°Brix) were determined in the clarified juices. One mL of each clarified juice was placed in the refractometer HI96811 (HANNA, Smithfield, RI, USA). Three samples of each clarified juice were used in this procedure.

### 3.4. Betacyanin and Betaxanthin Content and Antioxidant Activity Assay

#### 3.4.1. Quantification of Betacyanin and Betaxanthin

In order to determine the pulp’s betacyanin and betaxanthin content (µg/g, fresh weight) the spectrophotometric method described in [32,33,34] was carried out on clarified juices using a Model DR 500 spectrophotometer (Hach Lange GmbH, Düsseldorf, Germany). The clarified juices samples were diluted as shown in Table S2 in 5 mL volumetric flasks using Milli-Q water. The extinction coefficients used were E1% = 60,000 L mol^−1^ cm^−1^, λ = 540 nm for betacyanin, and E1% = 48,000 L mol^−1^ cm^−1^, λ = 480 for betaxanthin.

#### 3.4.2. Antioxidant Activity by 2,2’-Azino-bis-(3-ethylbenzothiazoline-6-sulfonic acid) Diammonium Salt Capacity (ABTS)

The ABTS, a single electron transfer (ET) reaction-based assay, was carried out following the method proposed by Re et al. [35]. The Phosphate buffered saline (PBS) used was created with 0.8 g of NaCl, 0.02 g of KH_2_PO_4_, 0.115 g of Na_2_HPO_4_, 0.02 g of KCl, and 0.02 g of NaN_3_. The volume was filled up to 100 mL, the difference being Milli-Q water. To create the ABTS reagent the following were used: 38.4 mg of ABTS 1 mM, 6.62 mg of potassium persulfate 2.45 mM, and 10 mL of the solution of PBS. The solutions were left mixing in the dark for 16 h. The absorbance was measured at 734 nm, with a spectrophotometer (Model DR 500, Hach Lange GmbH, Düsseldorf, Germany) and the dilution of the initial reagent that read 0.7 absorbance units was used (40 mL of PBS with 3 mL of ABTS solution). 20 µL of diluted clarified juice of each fruit and 2 mL of ABTS solution (with the absorbance of 0.7) were placed in a water bath at 30 °C for six minutes. Thereafter, the absorbance was read using Trolox as a standard in concentrations ranging from 5 to 200 ppm. The blank was created using Milli-Q water and the procedure was triplicated.

#### 3.4.3. Antioxidant Activity by α-α-Diphenyl-β-picrylhydrazyl (DPPH)

Based on Brand-Williams et al., protocol [36] 0.0148 g of DPPH were added to a 25 mL volumetric flask and filled to the mark with methanol (mother solution). One mL of this mother solution was added to a new 25 mL volumetric flask and filled to the mark with methanol, creating a diluted solution. The solutions were placed in 4.0 mL cuvettes (75 µL of clarified juices dilutions and 3 mL DPPH solution), and left to react for 16 min before being read by the spectrophotometer (Model DR 500, Hach Lange GmbH, Düsseldorf, Germany) at 515 nm [37]. All measurements were made in triplicates with a calibration curve of Trolox at varying concentrations from 5 to 200 ppm.

#### 3.4.4. Ferric Reducing Antioxidant Power (FRAP)

The antioxidant capacity of each clarified juice was determined through a modified method [38]. The FRAP reagent was prepared with acetate buffer 300 mM pH 3.6, which is a mixture of sodium acetate trihydrate, glacial acetic acid, and distilled water, a solution of iron triplicidyl triazine (TPTZ), concentrated HCl, and distilled water and finally a solution with FeCl_3_·6H_2_O and Milli-Q water. The solutions were mixed (10:1:1) respectively and incubated at 30 °C for 30 min in darkness. Then, 100 µL of each clarified juice was added to 3 mL of FRAP reagent. Concentrations of Trolox ranging from 10 to 200 ppm were used as standards in a calibration curve. Lastly, the absorbance was measured in a spectrophotometer (Model DR 500, Hach Lange GmbH, Düsseldorf, Germany) at 593 nm. All measurements were made in triplicate.

### 3.5. Total Phenolic Composition 

In order to determine the overall phenolic composition, the Folin–Ciocalteu colorimetric procedure was used [39]. Twenty µL of the diluted clarified juices were added to a 96 well plate. Subsequently, 100 µL of 10% Folin reagent was added to each well and after a five minutes incubation an additional 80 µL 7.5% w/v of sodium carbonate was placed per well. The plates were incubated for 1.5 h in the absence of light at 37 °C. Once the incubation period had elapsed, microplates were read at 765 nm and 25 °C. In order to create the calibration curve, solutions of 50 to 200 mg/L of gallic acid were made in Milli-Q water. The blank was created with the same solvent and the procedure was replicated twice (in triplicate).

### 3.6. HPLC-DAD Analysis

After filtering the clarified juices through a 0.2 µm nylon filter (Waters, Milford, MA, USA), their chromatographic profile was analyzed through equipment from Altus Perkin Elmer with an autosampler, photodiode array detector (PDA) and a Zorbax Eclipse XDB C18 column (5 μm, 150 × 4.6 mm). In order to analyze the phenolic composition, a gradient method was achieved with Solvent A, consisting of a mixture of water and acetic acid (pH 2.5), and Solvent B, methanol. The mobile phase composition started at 100% solvent A for 3 min, followed by an increase of solvent B up to 30% from minutes 3 to 8 min, 50% from minutes 8 to 15 min, 30% from 15 to 20 min, and then returning the mobile phase composition back to 100% solvent A for the end of the run. Around, 20 µL of the clarified juices were injected at a flow rate of 0.8 mL/min at column temperature of 25 °C. The UV absorption spectra were documented of clarified juices and standards at 270 nm. The phenolic acids, caffeic, gallic, p-coumaric, and vanillic acids were used as standards to compare retention time and identified compounds in the clarified juices. The standards were dissolved in milli-Q water to prepare the calibration curves.

### 3.7. Cell Viability Assay

#### 3.7.1. Cell Culture

A normal fibroblast cell line (NIH/ 3T3) and four different mammalian cancer cell lines: mammary (MCF-7), prostate (PC3), colon (Caco-2), and hepatic (HepG2), were cultivated in DMEM-F12 medium containing 10% FBS (Fetal Bovine Serum) (Gibco, Grand Island, NY, USA) and kept in a 5% CO_2_ atmosphere at 37 °C and 80% humidity.

#### 3.7.2. Cell Proliferation Assay

In order to determine viability, a cytotoxicity assay was carried out in 96-well plates with 100 µL of 5 × 10^5^ cells/mL per well. Cancer cell lines (MCF-7, PC3, Caco-2 and HepG2) and NIH/3T3 were seeded as a control and incubated for 12 h to reach confluence. All the clarified juices were evaluated at a final concentration, 2% *v*/*v*. The plates were left in the incubator at 37 °C, with less than 5% CO_2_ for 48 h. Subsequently, 20 µL of Cell Titer 96^®^AQueous One Solution Cell Proliferation Assay (Promega, Madison, WI) was added to each well, and the 96-well-plate was incubated at 37 °C, with 5% CO_2_ for 1 h, then the absorbance was read at 490 nm using a microplate reader (Synergy HT, Bio-Tek, Winooski, VM). The viability was determined through the calculation of average absorbance units per well and conveyed as a percentage of the untreated cell wells. The experiment was done in triplicate, culture medium without cells was used as a blank, and the cells that only had medium were a positive viability control [12].

### 3.8. Principal Component Analysis (PCA)

PCA is a tool used to highlight the relationships among a group of experimental variables based on multivariate statistical analysis, where a map is generated to show how variables are distributed. The correlation of antioxidant results and antiproliferative effect of the clarified juices of the cactus fruits was determined by inspection of the principal component analysis (PCA). The variables analyzed were percentage of cell viability on Caco-2, HepG2, PC3 and NIH/3T3 cell lines; antioxidant activity regarding DPPH (µmol TE/100 g FS), ABTS methods (µmol TE/100 g FS), total phenolic content obtained by the Folin–Ciocalteu method (µg GA/g of fresh sample) and betacyanin and betaxanthin (µg/g FS).

### 3.9. Statistical Analysis

All experiments were performed in triplicate. Results were analyzed by ANOVA and different means were compared using the Tukey test with a level of significance of *p* > 0.05. The computer software used was MINITAB 18. The multivariate analysis of Principal component analysis (PCA) above described was done by SPSS (Version 19, IBM Corp, Chicago, IL, USA.

## 4. Conclusions

The results presented in this paper prove the antioxidant properties of five Mexican native cactus fruits by ABTS, DPPH, FRAP and Folin–Ciocalteu methods, as well as the in vitro cell cytotoxicity on cell lines. Our findings exemplified the antiproliferative effect of *Myrtillocactus geomettizans* clarified juices through the diminished cell viability of Caco-2, HepG2, and PC3 as well as, on the normal cell line NIH/3T3. The red fruit *Stenocereus pruinosus* clarified juices affected the cancer cell line HepG2 as well as the NIH/3T3 cell line. *Opuntia ficus-indica* yellow fruit may potentially be used as a cancer preventing agent due to its selective cytotoxicity on only cancer cells, with demonstrated activity against HepG2 and no effect on the normal cell NIH/3T3. A future investigation may be performed in order to evaluate the time and dose-dependence of the cactus juices on cytotoxicity. The total phenolic compounds could be the main contributors of the bioactivity, although a synergistic effect between phenolic acids, flavonoids, coumarins, alkaloids, and vitamins could explain the cactus fruit juices’ cancer potential therapeutic use. More research is needed in order to identify the bioactive compounds and mechanisms that give the juices these properties. 

## Figures and Tables

**Table 1:** Betacyanin and betaxanthin content and antioxidant activity of clarified juices.

Parameter	SY	SR	OPY	OPR	MG
Betacyanins (µg/g FS)	47.99 ± 0.18 ^d^	334.06 ± 2.08 ^b^	13.29 ± 0.06 ^e^	403.56 ± 1.41 ^a^	103.50 ± 0.01 ^c^
Betaxanthins (µg/g FS)	240.52 ± 0.88 ^b^	404.59 ± 2.33 ^a^	59.28 ± 0.41 ^c^	263.24 ± 36.36 ^b^	45.76 ± 0.42 ^c^
Total phenolic compounds (mg GA/100 mL FS)	57.16 ± 1.52 ^e^	108.75 ± 0.35 ^c^	79.73 ± 1.04 ^d^	111.72 ± 0.35 ^b^	138.38 ± 0.14 ^a^
ABTS (µmol TE/100 g FS)	542.62 ± 7.20 ^d^	994.40 ± 32.28 ^c^	370.41 ± 10.69 ^e^	1097.35 ± 20.20 ^b^	3123.77 ± 26.15 ^a^
DPPH (µmol TE/100 mL FS)	80.71 ± 6.65	854.60 ± 17.60 ^b^	379.87 ± 70.86 ^c^	1115.25 ± 86.46 ^a^	329.24 ± 9.48 ^c^
FRAP (µmol TE/100 mL FS)	865.20 ± 10.24 ^c^	2744.48 ± 42.16 ^a^	480.20 ± 7.80 ^d^	2532.05 ± 48.63 ^b^	ND

Values represented as mean ± standard deviation (*n* = 3), different lowercase letters (**a–d**) indicate statistical significance difference (*p* < 0.05), FS = fresh sample, GA gallic acid equivalents, TE Trolox equivalents. ND = not determined *Stenocereus pruinosus* yellow fruit (SY), *Stenocereus pruinosus* red fruit (SR), *Opuntia ficus-indica* yellow fruit (OPY), *Opuntia ficus-indica* red fruit (OPR), and *Myrtillocactus geomettizans* fruit (MG). 2,2’-Azino-bis-(3-ethylbenzothiazoline-6-sulfonic acid) (ABTS), α-α-Diphenyl-β-picrylhydrazyl (DPPH), Ferric Reducing Antioxidant Power (FRAP.

**Table 2:** Phenolic acids’ composition of *Myrtillocactus geomettizans* fruit (MG) and *Opuntia ficus-indica* yellow fruit (OPY) detected by HPLC.

Parameter	MG Fruit mg/L FS	OPY Fruit mg/L FS
*p*-Coumaric acid	60.60 ± 0.25 ^a^	16.85 ± 1.02 ^b^
Gallic acid	14.95 ± 0.01 ^a^	21.75 ± 0.75 ^a^
Caffeic acid	1.90 ± 0.01 ^b^	4.60 ± 0.01 ^a^
Vanillic acid	10.05 ± 0.04 ^a^	13.00 ± 0.45 ^a^

Values represented as mean ± standard deviation (*n* = 3), different lowercase letters (**a,b**) indicate statistical significance difference (*p* < 0.05). FS = fresh sample.

## Data Availability

All data, belongs to this work, is given and presented herein the manuscript. Additional data can be found in the supplementary material.

## Supplementary Materials

The following are available online at https://www.mdpi.com/2223-7747/10/2/368/s1, Table S1: Total soluble solids (°Brix) in clarified juices, Table S2: Dilutions of the clarified juices in the different techniques, Table S3: Calculated concentration of phenolic acids composition of *Myrtillocactus geomettizans* fruit (MG) and *Opuntia ficus-indica* yellow fruit (OPY) detected by HPLC. Figure S1: General procedure to obtain clarified juices from cactus fruits Figure S2: HPLC detection of phenolic compounds (vanillic acid and coumaric acid) from *Myrtillocactus geomettizans* fruit (MG), Figure S3: HPLC detection of phenolic compounds (gallic acid and caffeic acid) from *Myrtillocactus geomettizans* fruit (MG), Figure S4: HPLC detection of phenolic compounds (vanillic acid and coumaric acid) from *Opuntia ficus-indica* yellow fruit (OPY), Figure S5: HPLC detection of phenolic compounds (gallic acid and caffeic acid) from *Opuntia ficus-indica* yellow fruit (OPY).
