# Supplementary material for: Underutilized Mexican Plants: Screening of Antioxidant and Antiproliferative Properties of Mexican Cactus Fruit Juices
Source: Plants (Basel). 2021 Feb 14;10(2):368. doi: 10.3390/plants10020368 (PMC7918198; doi:10.3390/plants10020368)
Supplement: Supplementary file 1 [file plants-10-00368-s001.pdf]

Supplementary material

Research Article

Underutilized Mexican Plants: Screening of Antioxidant and Antiproliferative Properties of Mexican Cactus Fruits Juices

Elda M. Melchor Martínez, Luisaldo Sandate-Flores, José Rodríguez-Rodríguez, Magdalena Rostro-Alanis, Lizeth Parra-Arroyo, Marilena Antunes-Ricardo, Sergio O. Serna-Saldívar, Hafiz M.N. Iqbal* and Roberto Parra-Saldívar *

1 Tecnologico de Monterrey, School of Engineering and Sciences, Monterrey, 64849 México; elda.melchor@tec.mx, a00812589@itesm.mx, jrr@tec.mx, magda.rostro@tec.mx, a01036078@itesm.mx, marilena.antunes@tec.mx, sserna@tec.mx.

* Correspondence: hafiz.iqbal@tec.mx (H.M.N.I), r.parra@tec.mx (R.P.S)

Table S1. Total soluble solids (°Brix) in clarified juices

Clarified juices	°Brix
SY	0.2±0.0
SR	0.2±0.0
OPY	0.1±0.0
OPR	0.1±0.0
MG	0.1±0.0

- The values are represented as mean ± standard deviation (n = 3), S*tenocereus pruinosus* yellow fruit (SY), S*tenocereus pruinosus* red fruit (SR), *Opuntia ficus-indica* yellow fruit (OPY), *Opuntia ficus-indica* red fruit (OPR), and *Myrtillocactus geomettizans* fruit (MG). The total solids were used to adapt units (assuming a density equal to water 1 kg/L) and compare results against the literature.

Table S2. Dilutions of the clarified juices in the different techniques.

Parameter	SY	SR	OPY	OPR	MG
Betacyanins (μg/g FS)	1:25	1:25	1:5	1:25	1:5
Betaxanthins (μg/g FS)	1:25	1:25	1:10	1:25	1:5
ABTS (μmol TE/100 g FS)	1:5	1:10	1:5	1:10	1:25
DPPH μmol TE / 100 mL FS)	1:25	1:25	1:10	1:25	1:5
FRAP (μmol TE / 100 mL FS)	1:25	1:25	1:10	1:25	ND
Total phenolic compounds (mg GA/100 mL FS)	1:25	1:25	1:5	1:25	1:5

Values represent the dilutions used. FS= fresh sample, GA gallic acid equivalents, TE Trolox equivalents. ND = not determined S*tenocereus pruinosus* yellow fruit (SY), S*tenocereus pruinosus* red fruit (SR), *Opuntia ficus-indica* yellow fruit (OPY), *Opuntia ficus-indica* red fruit (OPR), and *Myrtillocactus geomettizans* fruit (MG). The clarified juices of MG and OPY analyzed by HPLC were diluted 1: 5.

Table S3 Calculated concentration of phenolic acids composition of *Myrtillocactus geomettizans* fruit (MG) and *Opuntia ficus-indica* yellow fruit (OPY) detected by HPLC

**Phenolic acid**	**mg/L of fresh sample of MG fruit detected by HPLC analysis**	**Calculated concentration of phenolic acids in the clarified juice at 2% (v/v) of MG used at cytotoxic assay (μg/100 μL)**	**mg/L of fresh sample of OPY fruit detected by HPLC analysis**	**Calculated concentration of phenolic acids in the clarified juice at 2 %(v/v) of OPY used at cytotoxic assay (μg/100 μL)**
***p*-coumaric acid**	60.60 ± 0.25^{a}	0.120 ± 0.25^{a}	16.85 ± 1.02^{b}	0.030 ± 1.02^{b}
**Gallic acid**	14.95 ± 0.01^{a}	0.030 ± 0.01^{a}	21.75 ± 0.75^{a}	0.040 ± 0.75^{a}
**Caffeic acid**	1.90 ± 0.01^{b}	0.004 ± 0.01^{b}	4.60 ± 0.01^{a}	0.009 ± 0.01^{a}
**Vanillic acid**	10.05 ± 0.04^{a}	0.020 ± 0.04^{a}	13.00 ± 0.45^{a}	0.030 ± 0.45^{a}

Values represented as mean ± standard deviation ($n = 3$), different lowercase letters (a-b) indicate statistical significance difference ($p < 0.05$).

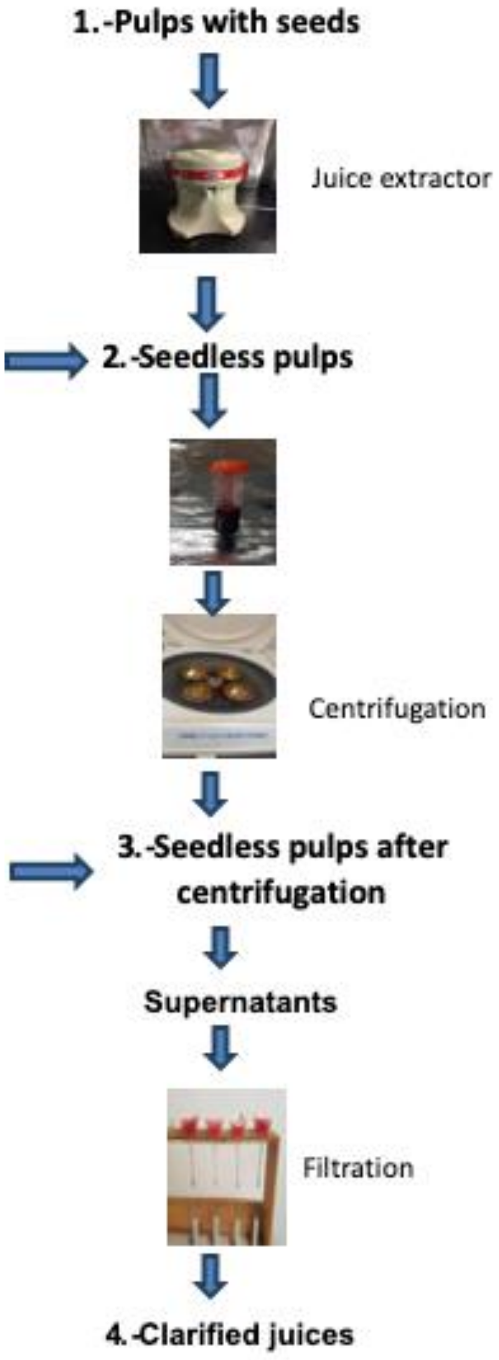

Figure S1. General procedure to obtain clarified juices from cactus fruits.

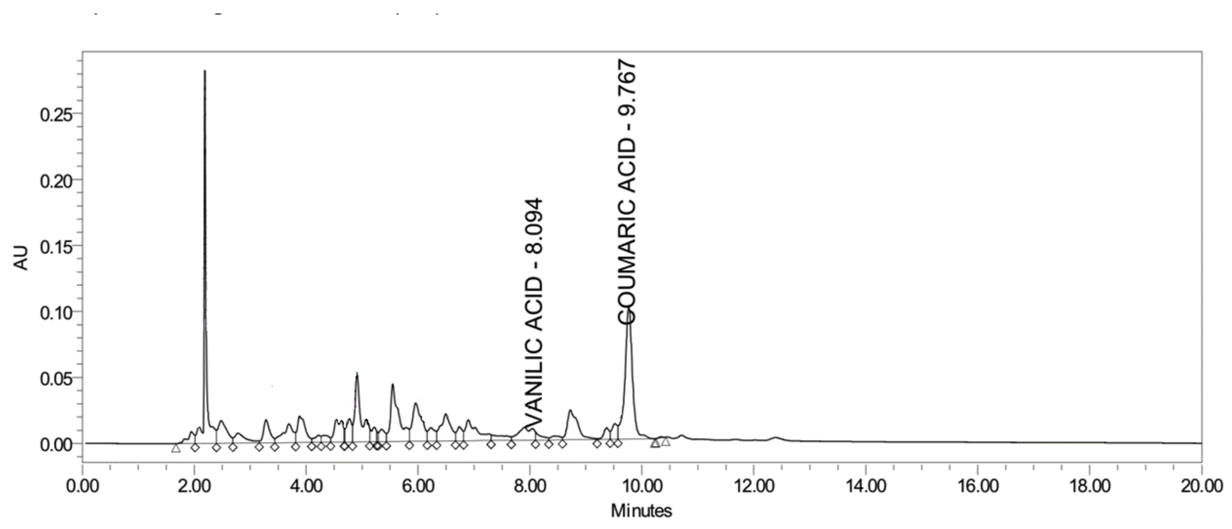

Figure S2. HPLC detection of phenolic compounds (vanillic acid and coumaric acid) from *Myrtillocactus geomettizans* fruit (MG).

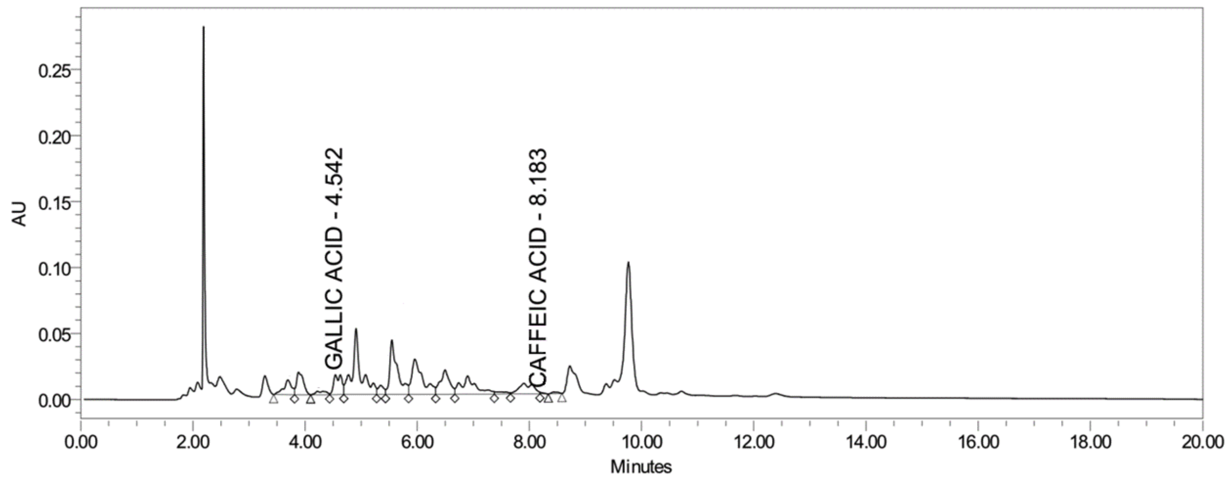

Figure S3. HPLC detection of phenolic compounds (gallic acid and caffeic acid) from *Myrtillocactus geomettizans* fruit (MG).

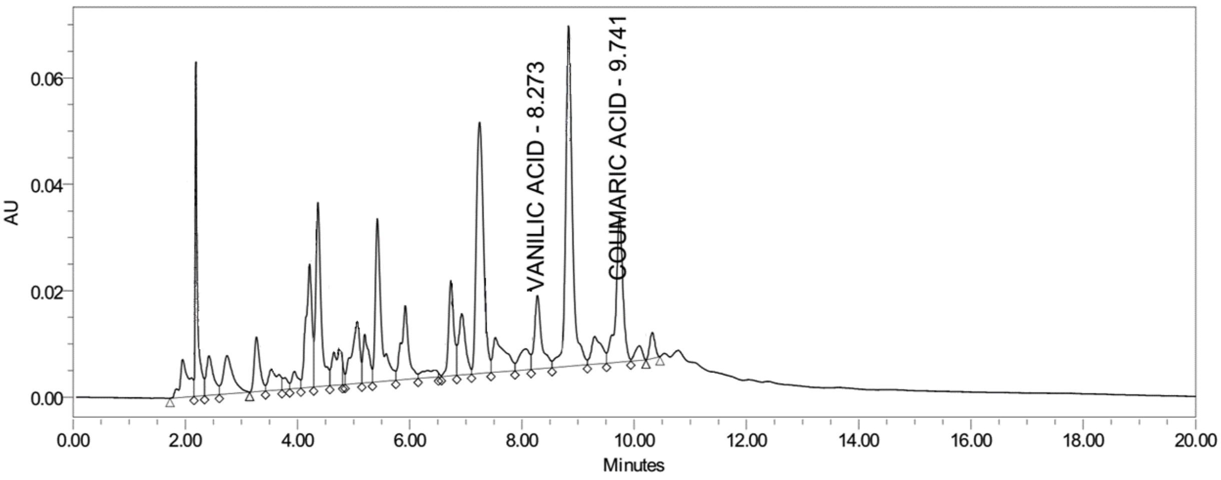

Figure S4. HPLC detection of phenolic compounds (vanillic acid and coumaric acid) from *Opuntia ficus-indica* yellow fruit (OPY).

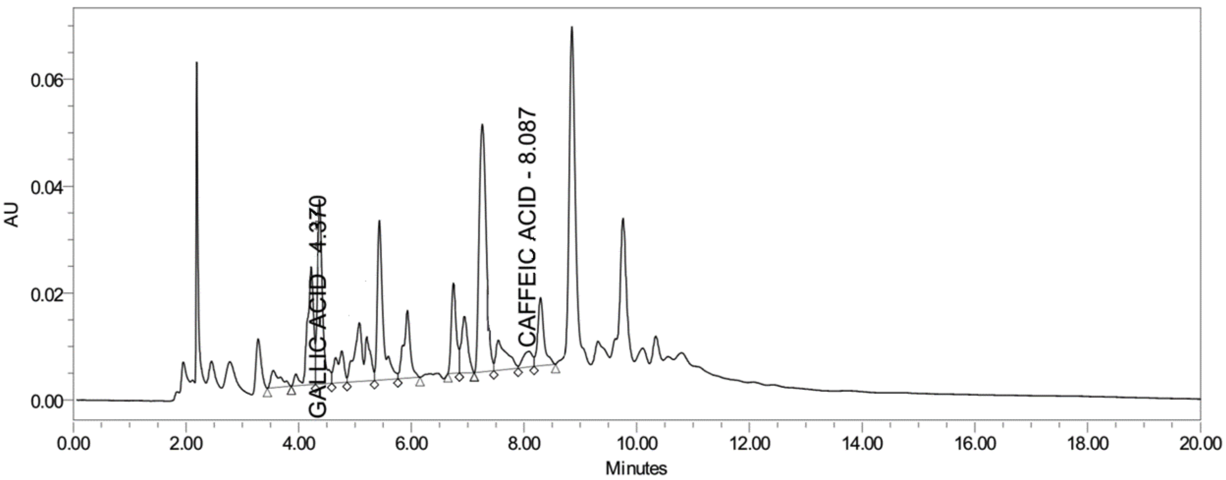

Figure S5. HPLC detection of phenolic compounds (gallic acid and caffeic acid) from *Opuntia ficus-indica* yellow fruit (OPY).
